# Bilateral Obstructive Uropathy Requiring Hemodialysis With Presumed Spontaneous Stone Clearance After Two Years of Forgotten Ureteral Stents: A Case Report

**DOI:** 10.7759/cureus.111269

**Published:** 2026-06-22

**Authors:** Hasan F Buali, Tarek Abushloa, Khaled Ahmed

**Affiliations:** 1 Urology, King Hamad University Hospital, Muharraq, BHR

**Keywords:** acute kidney injury, bilateral ureteral obstruction, double-j stent, forgotten ureteral stent, obstructive uropathy, spontaneous stone passage, ureteral calculi

## Abstract

Bilateral ureteral obstruction from urolithiasis is an uncommon but life-threatening cause of obstructive acute kidney injury (AKI). Double-J (DJ) stent insertion achieves emergent bilateral decompression and allows elective definitive stone management; however, the recommended stent dwell time varies by stent material and patient risk factors, as encrustation risk rises substantially after a few weeks to months of indwelling time. We report the case of a 78-year-old male with chronic kidney disease (CKD) who presented with bilateral proximal ureteral stones, severe AKI requiring hemodialysis, and a coexisting bladder stone. He was managed with bilateral DJ stent insertion and discharged, but was subsequently lost to follow-up for two years due to international travel. On return, both ureteral stones had undergone presumed spontaneous passage, confirmed by radiographic resolution on CT. Both stents were retrieved without complication, and no encrustation was identified. The persistent bladder stone was managed by cystolithotripsy at the same setting. Renal function at the two-year return visit remained stable relative to discharge, consistent with partial recovery and persistent advanced CKD. This report highlights the possibility of presumed spontaneous proximal ureteral stone clearance under prolonged stenting, the importance of structured stent follow-up systems, and the unexpectedly favorable outcomes achievable even with prolonged stent retention in the absence of documented urinary tract infection.

## Introduction

Bilateral ureteral obstruction leading to acute kidney injury (AKI) represents a urological emergency that is infrequently caused by benign stone disease and requires urgent recognition and management [1]. Prompt decompression using ureteral stenting or percutaneous nephrostomy relieves obstruction and facilitates renal recovery, while definitive stone treatment is typically deferred to an elective setting [1,2]. Renal functional recovery following relief of bilateral obstruction is influenced by the severity and duration of the obstruction, the degree of pre-existing renal impairment, and the presence of concurrent infection [2]. In a large multicenter cohort of patients with obstructive uropathy, 78% presented with AKI and 16.4% required temporary dialysis, with pre-existing chronic kidney disease (CKD) identified as an independent risk factor for both the development of AKI and the failure of short-term renal recovery [3].

The recommended dwell time for a double-J (DJ) stent, a flexible endoscopically placed tube designed to maintain ureteral patency and facilitate urine drainage, varies depending on stent material, patient risk factors, and clinical indication, but generally ranges from a few weeks to a maximum of six months, after which encrustation risk rises substantially [4]. Beyond this window, the risk of encrustation increases proportionally with the duration of indwelling, driven by urinary crystal deposition on the stent surface, bacterial biofilm formation, and polymer degradation [4,5,6]. Progressive encrustation can lead to complete ureteral obstruction, renal functional deterioration, and, in severe cases, irreversible loss of the affected kidney [4,5,6].

When a stent becomes heavily encrusted, simple cystoscopic removal is no longer feasible, and the encrustation must first be fragmented using stone-clearing techniques before the stent can be extracted. Depending on the location and burden of encrustation, these techniques include extracorporeal shock wave lithotripsy, ureteroscopic laser lithotripsy, cystolitholapaxy, and percutaneous nephrolithotomy; in extreme cases, particularly when the affected kidney is non-functional and the stent is extensively adherent, nephroureterectomy has been described as a definitive management option [4-6].

In addition to the risk of encrustation, prolonged DJ stent retention is also commonly associated with stent-related symptoms, including urinary frequency, urgency, dysuria, flank pain, and hematuria, which often prompt patients to seek earlier removal [7]. Presumed spontaneous stone passage (SSP) under an indwelling ureteral stent is an established but unpredictable phenomenon, reported in approximately 24% of stented patients within four weeks of stent placement [8]. SSP is most commonly observed in distal ureteral stones at the ureterovesical junction and least commonly in proximal ureteral stones, with stone size, location, and density identified as independent predictors [8]. Notably, proximal ureteral stones larger than 7 mm are associated with the lowest likelihood of spontaneous passage in this setting [8].

We present a case in which bilateral proximal ureteral stones causing dialysis-requiring AKI were managed with bilateral DJ stenting, followed by unplanned stent retention for two years due to patient travel. On return, both stones were no longer visible on CT, both stents were removed without complication, and no encrustation was identified, representing an unusual constellation of outcomes in the context of prolonged stent retention.

## Case presentation

A 78-year-old male with known CKD (baseline serum creatinine approximately 240 µmol/L, consistent with CKD stage 4), hypertension, and a large prostate presented to the emergency department on November 28 with a one-week history of worsening bilateral flank pain that was associated with nausea and eight hours of oliguria. He denied vomiting or fever. Bladder catheterization yielded only 10 mL, suggesting that lower urinary tract retention was unlikely. A point-of-care bedside ultrasound performed by the emergency department team raised concerns for bilateral hydronephrosis; this image was not stored. Laboratory investigations on admission are summarized in Table 1.

**Table 1:** Laboratory investigations on admission ECG: electrocardiograph

Parameter	Patient value	Reference range
Serum creatinine (µmol/L)	1134.9	62-115
Serum potassium on admission (mmol/L)	6.8	3.5-5.1
Serum potassium post-dialysis (mmol/L)	4.2	3.5-5.1
Urea (mmol/L)	49.3	3.7-7.0
Hemoglobin (g/dL)	9.7	13.5-17.5
White blood cell count (x10⁹/L)	3.3	4.0-11.0
Arterial pH	7.131	7.35-7.45
Bicarbonate (mmol/L)	8.2	22-29
Urinalysis - red blood cells (per high-power field)	Greater than 50	0-3
Urinalysis - white blood cells (per high-power field)	5-10	0-5
Urine culture	Negative	Negative
ECG	Normal sinus rhythm	Normal

The patient was admitted to the ICU with a diagnosis of AKI secondary to bilateral obstructive uropathy, complicated by acidosis (pH 7.131, bicarbonate 8.2 mmol/L) and hyperkalemia. Anti-hyperkalemic measures were instituted; however, given the severity of the metabolic derangements and the failure to achieve adequate correction with medical therapy alone, hemodialysis was initiated on the same day of admission (November 28). A further session was completed on November 29. Decompression was intentionally deferred until hemodynamic stabilization was achieved following these dialysis sessions. Once the patient was clinically stable on November 30, a non-contrast CT of the kidneys, ureters, and bladder (CT KUB) was performed, demonstrating bilateral proximal ureteral stones (left 10 × 5.4 mm; right 8 × 6.1 mm; average stone density 400 HU) with bilateral moderate hydronephrosis and hydroureter, bilateral renal parenchymal thinning consistent with underlying CKD, and a 9 mm bladder stone (Figures 1, 2). Stone density below 1000 HU is associated with higher rates of presumed spontaneous passage under ureteral stenting [8].

**Figure 1:**
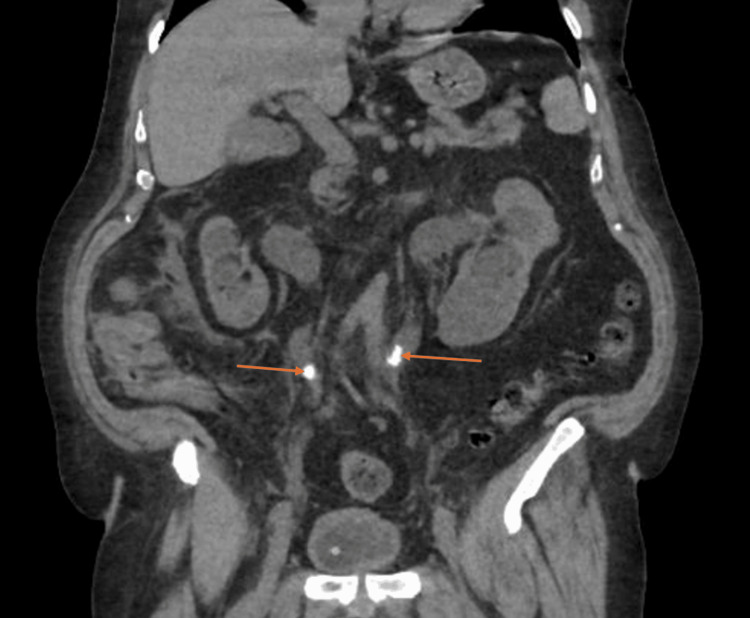
Non-contrast CT demonstrating bilateral proximal ureteral stones 10 × 5.4 mm (left) and 8 × 6.1 mm (right), with bilateral moderate hydronephrosis and hydroureter CT: computed tomography

**Figure 2:**
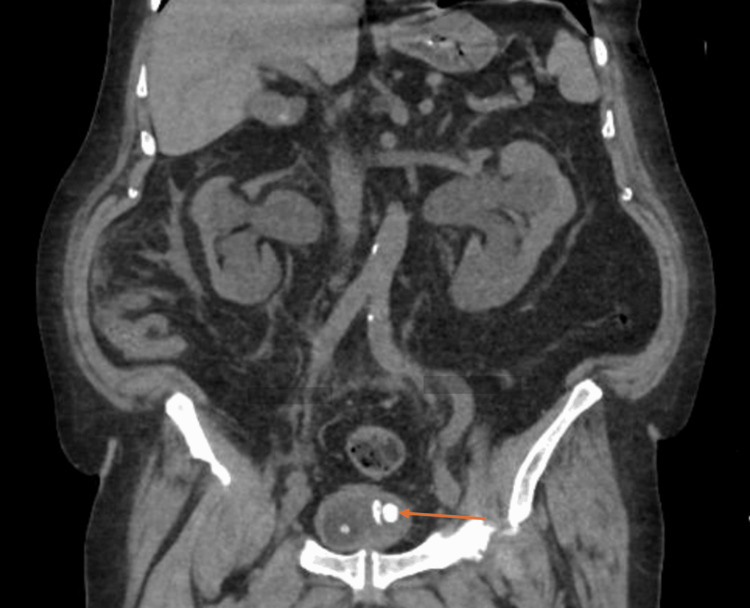
Non-contrast CT demonstrating the urinary bladder stone (9 mm) CT: computed tomography

Following stabilization, the patient was taken to the operating theater on the same day. Cystoscopy confirmed a bladder stone, and the bilateral ureteral orifices were identified with difficulty owing to the large median lobe. Bilateral 6 Fr, 26 cm DJ stents were successfully inserted. Cystolithotripsy was deferred during this procedure, given the patient's critical condition and the primary goal of achieving emergent ureteral decompression. The bladder stone was planned for elective management at a subsequent visit. The serum creatinine trended down to 461.8 µmol/L at discharge, and the serum potassium normalized to 4.4 mmol/L. The patient was instructed to attend outpatient urology follow-up for definitive stone management and elective stent exchange; however, he traveled internationally and was lost to follow-up.

Two years later, the patient self-presented to the urology outpatient clinic. He remained clinically well and reported no urinary tract symptoms. The preoperative urine culture was negative. Serum creatinine was 472.3 µmol/L, and serum potassium was 5.1 mmol/L. Repeat non-contrast CT of the kidneys, ureters, and bladder (CT KUB) demonstrated complete resolution of the bilateral hydronephrosis and hydroureter. Both previously seen ureteral stones were no longer visible on repeat CT, suggesting their presumed spontaneous passage. Persistent bilateral renal parenchymal thinning, a residual 6 mm calculus in the bladder lumen, and bilateral DJ stents in situ were also noted (Figure 3). The bladder stone was considered primary in etiology, most likely related to urinary stasis secondary to bladder outlet obstruction from the large prostate, as it was already present on the initial CT at a time when both ureteral stones remained in situ. Surgical intervention for the enlarged prostate was not indicated at either presentation, as the patient reported voiding freely without lower urinary tract complaints throughout.

**Figure 3:**
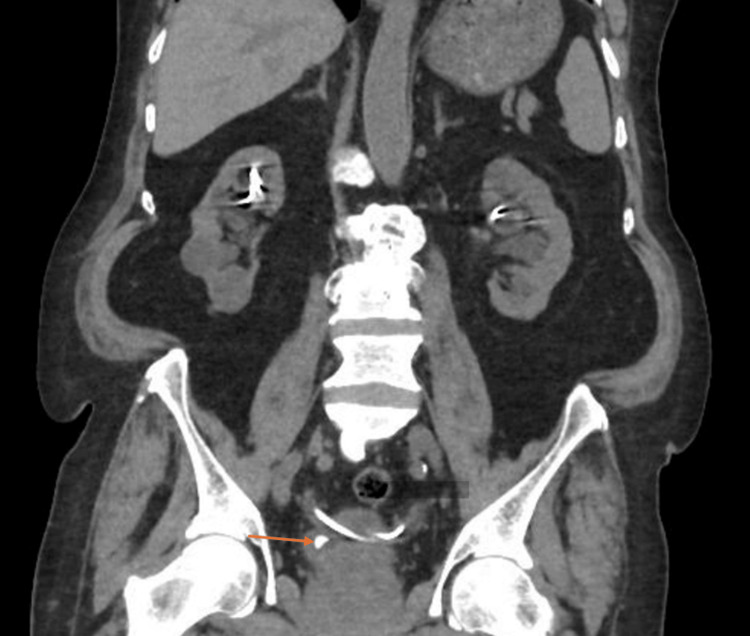
Non-contrast CT demonstrating complete resolution of the bilateral hydronephrosis Both previously seen ureteral stones are no longer visible, suggesting presumed spontaneous passage, a persistent 6 mm bladder calculus, and bilateral DJ stents in situ

The patient underwent cystolithotripsy for the residual bladder stone, followed by bilateral cystoscopic stent removal. Both stents were extracted without resistance. Visual inspection confirmed no macroscopic encrustation or fragmentation of either stent (Figure 4). The stents were visually consistent with polyurethane composition, demonstrating discoloration consistent with prolonged indwelling time but no calcific deposits. Encrustation in polyurethane stents is strongly associated with bacterial biofilm formation, which is consistent with the absence of documented urinary tract infection in this case [4]. He was discharged and remained clinically stable at subsequent outpatient review.

**Figure 4:**
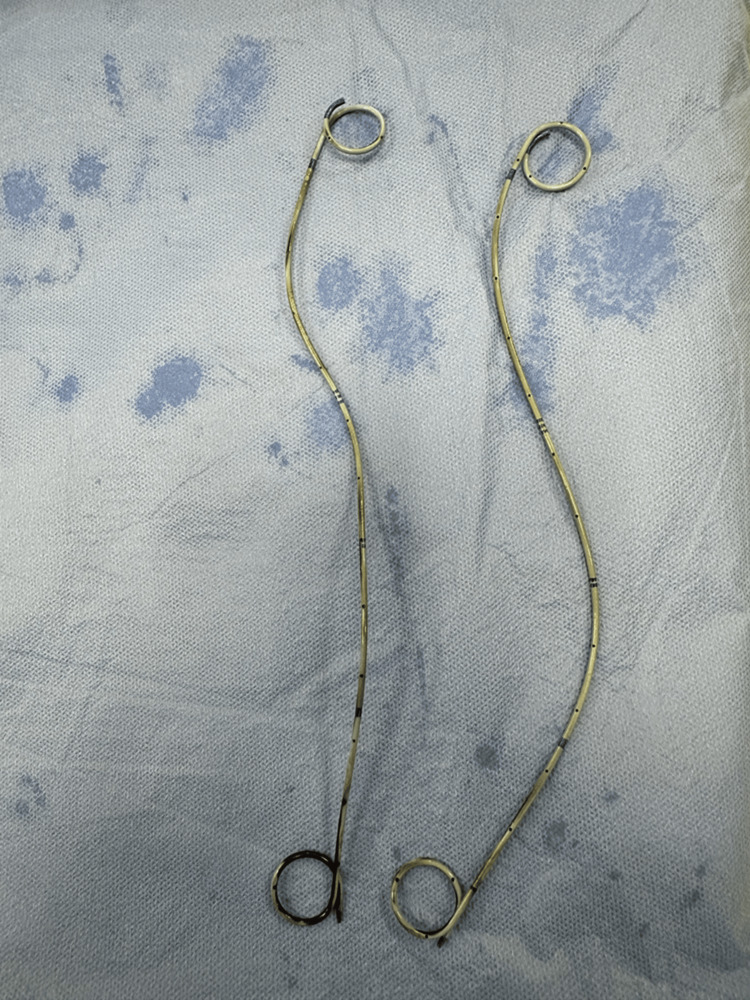
The two retrieved double-J ureteral stents following cystoscopic removal after two years of indwelling time

## Discussion

This case report presents two clinically distinct and equally instructive findings: the presumed spontaneous passage of bilateral proximal ureteral stones under prolonged DJ stenting, and the uncomplicated retrieval of stents retained for two years without encrustation. Bilateral ureteral obstruction from urolithiasis requiring hemodialysis is an uncommon presentation, as most patients with bilateral stone disease retain sufficient function to avoid dialysis-level AKI [1]. In our case, the combination of pre-existing CKD stage 4 (baseline creatinine approximately 240 µmol/L) and simultaneous bilateral proximal obstruction created a scenario in which decompression alone, without concurrent active stone intervention, was the appropriate immediate strategy. Hemodialysis was initiated first to correct the life-threatening metabolic derangements, with ureteral decompression deferred until hemodynamic stability was achieved - a sequence consistent with established management principles for critically ill patients with obstructive uropathy [1,3].

Renal functional recovery following relief of bilateral obstruction is determined by multiple factors, including the duration and degree of obstruction and the pre-existing renal reserve [2]. A large multicenter cohort similarly found that pre-existing CKD was the only independent predictor of failed short-term renal recovery following decompression of obstructive uropathy, regardless of the underlying etiology [3]. In the present case, the serum creatinine improved from 1134.9 µmol/L at presentation to 461.8 µmol/L at discharge and remained stable at 472.3 µmol/L at the two-year return visit. It is acknowledged that this value represents persistent severe renal impairment; the stability observed reflects a return toward the patient's pre-existing CKD baseline rather than complete renal recovery.

The presumed spontaneous passage of proximal ureteral stones measuring 10 × 5.4 mm and 8 × 6.1 mm represents one of two equally instructive findings in this case, the other being the absence of encrustation following two years of stent retention. Published data indicate that SSP in stented patients occurs in approximately 24% of cases overall, with stone location at the ureterovesical junction, distal ureteral location, size ≤5 mm, and low stone density representing independent predictors of passage [8]. SSP rates for proximal ureteral stones are substantially lower than for distal stones, approaching zero at the ureteropelvic junction in some series [8]. In the present case, the average stone density of 400 HU - below the 1000 HU threshold associated with higher SSP probability - may have been a contributing factor to the radiographic resolution observed [8]. The mechanism by which prolonged stenting may facilitate proximal stone passage likely involves passive ureteral dilatation over time; a DJ stent abolishes normal ureteral peristalsis and induces loss of ureteral coaptation, gradually widening the ureteral lumen and potentially allowing a stone to migrate distally alongside the stent shaft - a process that may be accelerated over a two-year indwelling period compared to the standard four-week observation window [8].

Both the American Urological Association (AUA) and European Association of Urologists (EAU) guidelines recommend active stone removal via ureteroscopy or shock wave lithotripsy for proximal ureteral stones exceeding 10 mm, particularly in the presence of obstruction or impaired renal function [9]. In the present case, the left stone (10 × 5.4 mm) met this threshold; however, the patient's clinical course rendered active intervention unnecessary, illustrating that guideline-based thresholds represent population-level recommendations rather than absolute requirements for every individual.

The presumed passage of both proximal stones in this case, over a two-year interval under passive stent-facilitated ureteral dilatation and without any additional active intervention, illustrates that SSP in stented patients can occur with stones that fall outside conventional expulsive therapy thresholds and would ordinarily be managed by ureteroscopy or extracorporeal shock wave lithotripsy. It should be noted that the stone passage was not directly witnessed, and radiographic disappearance on CT remains the sole basis for this conclusion; impaction status and stone composition were unavailable as the stones were not retrieved, representing a limitation of this report.

The absence of stent encrustation following two years of retention is the most remarkable finding. Encrustation is the defining complication of prolonged stent retention [4]. Published case reports describe severe encrustation with stents retained for comparable or shorter periods, with reported outcomes including nephroureterectomy for a non-functioning kidney after 25 years of retention [4], an emphysematous perinephric abscess after 10 years of retention [5], and complex multimodal endourological and open surgical procedures for a stent retained for 15 years [6]. In each of these cases, infection, particularly with ureolytic bacteria capable of raising urinary pH and promoting struvite crystal deposition, was a key driver of encrustation. The stents were visually consistent with polyurethane composition; encrustation in polyurethane stents is strongly associated with bacterial biofilm formation, which is consistent with the absence of documented urinary tract infection in this case [4,5,6].

The absence of documented urinary tract infection at both the initial presentation and the two-year return visit may have contributed to the lack of encrustation observed; it is acknowledged that negative point-in-time cultures do not exclude subclinical biofilm formation during the two-year interval, and this represents an inherent limitation of the available data. A detailed metabolic profile and comprehensive urinary chemistry were not performed in this case, which represents a further limitation. The clinical lesson is that, while encrustation is expected with prolonged retention, it is not universal and may be substantially mitigated by the absence of urinary tract infection.

It is also notable that the patient remained entirely asymptomatic throughout the two-year retention period. Prolonged DJ stent retention is frequently associated with bothersome lower urinary tract symptoms, including urinary frequency, urgency, dysuria, and flank pain, which commonly prompt patients to seek earlier medical attention and stent removal [7]. The absence of such symptoms in this case likely contributed to the prolonged, unplanned retention, as the patient had no clinical trigger to seek follow-up despite traveling abroad.

This case also highlights the preventable nature of unplanned stent retention. Structured stent registry systems, automated patient reminder programs, and coordinated discharge planning - particularly for patients with known international travel plans - represent evidence-supported strategies for reducing the incidence of forgotten stents [4]. In our case, the absence of stent-related symptoms removed any patient-driven impetus for follow-up, reinforcing the need for institution-led tracking systems that operate independently of patient symptomatology. At our institution, SMS reminders are routinely sent to patients to alert them of upcoming appointments; however, in this case, the patient's international travel and change of contact availability rendered this measure ineffective, highlighting that reminder systems alone are insufficient without a dedicated ureteral stent registry in which all insertions are logged with scheduled removal dates and escalation protocols for non-responders. Although the outcome in the present case was favorable, the potential consequences of prolonged unmonitored retention are severe, and systematic measures to prevent loss to follow-up remain a clinical priority.

## Conclusions

We described a case of radiographic resolution of bilateral proximal ureteral stones following two years of unplanned DJ stent retention, with uncomplicated bilateral stent removal and no evidence of encrustation. The favorable outcome is at least partly attributable to the absence of documented urinary tract infection during the entire retention period. This report illustrates the potential for presumed spontaneous passage of proximal ureteral stones under prolonged stenting, underscores the importance of structured stent follow-up systems to prevent unplanned retention, and demonstrates that encrustation, although expected with prolonged indwelling time, is not inevitable in the absence of urinary tract infection.
